# Supplementary material for: Application of Exploratory Factor Analysis and Item Response Theory to Validate NHANES ADL Scale in Patients Reporting Rheumatoid Arthritis
Source: Pharmacy (Basel). 2022 Oct 22;10(6):138. doi: 10.3390/pharmacy10060138 (PMC9680378; doi:10.3390/pharmacy10060138)
Supplement: Supplementary file 1 [file pharmacy-10-00138-s001.zip › pharmacy-1909022-supplementary.pdf]

Figure S1. Path Diagram.

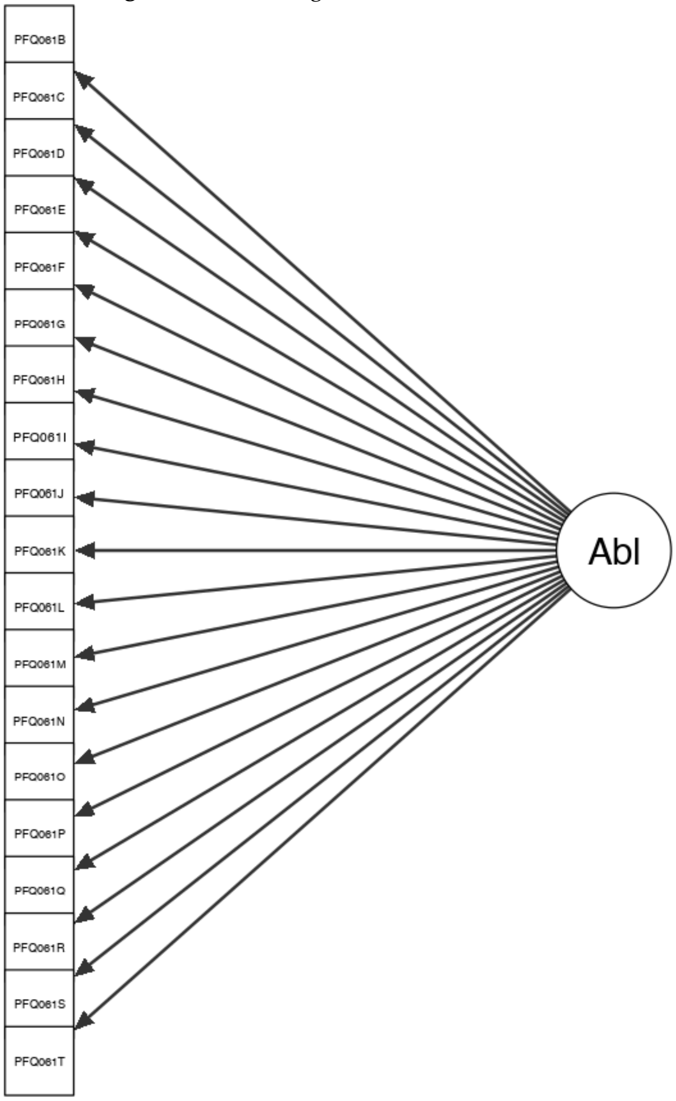

Abl: Ability; PFQ061B- PFQ061T: NHANES ADL Items 1 to 19

Table S1. The NHANES-ADL Scale and Item Means.

Item	Item mean	SD
Difficulty walking for a quarter mile	1.48	.741
Difficulty walking up ten stairs	1.34	.621
Difficulty stooping, crouching, kneeling	1.80	.820
Difficulty lifting or carrying	1.45	.759
Difficulty doing house chores	1.30	.533
Difficulty preparing meals	1.10	.330
Difficulty walking between rooms	1.04	.214
Difficulty standing up from armless chair	1.22	.476
Difficulty getting in and out of bed	1.21	.457
Difficulty using fork, knife, cup	1.05	.230
Difficulty dressing yourself	1.17	.395
Difficulty standing for long periods	1.76	.867
Difficulty sitting for long periods	1.41	.675
Difficulty reaching up	1.34	.610
Difficulty grasp/holding small objects	1.28	.530
Difficulty going out to movies/events	1.25	.502
Difficulty attending social event	1.22	.509
Difficulty with home leisure activities	1.07	.282
Difficulty moving large objects	1.69	.891

SD: Standard Deviation.

Table S2. Model fit Measures.

**Test for Exact Fit**			**Fit Measures**				RMSEA, 90%CI	
$\chi 2$	df	p-value	CFI	TLI	SRMR	RMSEA	Lower	Upper
1863	150	<0.001	0.890	0.874	0.0510	0.0821	0.0788	0.0855

$\chi 2$: Chi-squared; df: Degrees of Freedom; CFI: Comparative Fit Index; TLI: Tucker Lewis Index; SRMR: Standardized Root Mean Square Residual; RMSEA: Root Mean Square Error of Approximation; CI: Confidence Interval.
